# RETRACTED: Ouyang et al. Precision Isolation of Circulating Leukemia Cells in Chronic Myelogenous Leukemia Patients Using a Novel Microfluidic Device and Its Clinical Applications. *Cancers* 2023, *15*, 5696

**DOI:** 10.3390/cancers17203332

**Published:** 2025-10-16

**Authors:** Dongfang Ouyang, Ningxin Ye, Kun Yang, Yiyang Wang, Lina Hu, Shuen Chao, Yonghua Li

**Affiliations:** 1 Center for Engineering in Medicine and Surgery, Massachusetts General Hospital, Harvard Medical School, Charlestown, Boston, MA 02129, USA; elaine8191@gmail.com; 2 Shriners Hospital for Children, Boston, MA 02114, USA; 3 Department of Biology, University of Ottawa, Ottawa, ON K1N 6N5, Canada; nye025@uottawa.ca; 4 Department of Mechanical & Industrial Engineering, University of Toronto, Toronto, ON M5S 3E8, Canada; kunn.yang@mail.utoronto.ca; 5 Department of Microbiology, Immunology and Molecular Genetics, University of California Los Angeles (UCLA), Los Angeles, CA 90095, USA; jenniferwang366@gmail.com; 6 Department of Hematology, Shenzhen People’s Hospital, Shenzhen 518020, China; ourgba2020@gmail.com; 7 Department of Hematology, PLA General Hospital of Southern Theater Command, Guangzhou 510010, China

The journal retracts the article entitled “Precision Isolation of Circulating Leukemia Cells in Chronic Myelogenous Leukemia Patients Using a Novel Microfluidic Device and its Clinical Applications” [1].

Following publication, concerns were brought to the attention of the Editorial Office regarding the authenticity of the authorship list and the origins of this study [1].

Adhering to our standard procedure, an investigation was conducted by the Editorial Office and Editorial Board, who were unable to verify the identity, contribution, or affiliations of a number of the authors listed on this manuscript. Further concerns relating to the origins of this study could not be adequately resolved. As a consequence, the Editorial Board has lost confidence in the integrity of the findings and has decided to retract this study [1] as per MDPI’s retraction policy (https://www.mdpi.com/ethics#_bookmark30, accessed on 16 September 2025) and amend the original authorship list to remove an author that was confirmed to have no knowledge of connection to this study.

This retraction and amendment to the original authorship list was approved by the Editor-in-Chief of the journal *Cancers*.

Yonghua Li disagreed with this retraction; the other authors did not comment on this decision.
